# Acute severe cholestatic hepatitis and lymphopenia characterize pediatric hepatitis‐associated aplastic anemia

**DOI:** 10.1002/jpn3.70308

**Published:** 2025-12-09

**Authors:** Daniel Tegtmeyer, Sofia Tsaka, Kai Lehmberg, Michaela Höfs, Jan Beime, Andrea Briem‐Richter, Jun Oh, Elke Lainka, Sebastian Schulz‐Jürgensen

**Affiliations:** ^1^ Department of Pediatric Nephrology, Hepatology and Transplantation University Children's Hospital, University Medical Center Hamburg‐Eppendorf Hamburg Germany; ^2^ German Center for Child and Adolescent Health (DZKJ) Hamburg Germany; ^3^ European Reference Network for Hepatological Diseases (ERN RARE‐LIVER) Hamburg Germany; ^4^ Department of Paediatrics II University Children's Hospital, Pediatric Gastroenterology, Hepatology, and Transplant Medicine, University Duisburg‐Essen Essen Germany; ^5^ Department of Pediatric Hematology and Oncology, Division of Pediatric Stem Cell Transplantation and Immunology University Children's Hospital, University Medical Center Hamburg‐Eppendorf Hamburg Germany; ^6^ Department of Pediatrics III University Children's Hospital, Pediatric Hematology and Oncology, University Duisburg‐Essen Essen Germany

**Keywords:** acute liver failure, acute severe hepatitis, bone marrow failure

## Abstract

**Objectives:**

Hepatitis‐associated aplastic anemia (HAAA) is described as acute severe hepatitis of unknown origin followed by bone marrow failure (BMF). We aimed to provide a comprehensive picture of pediatric HAAA.

**Methods:**

Two‐center retrospective analysis was performed using data from children diagnosed with acquired BMF, including severe aplastic anemia (SAA) and myelodysplastic syndrome type refractory cytopenia of childhood (RCC). The assessment of the subcohort of HAAA included clinical features indicative of diagnosis and disease progression, with additional data from previously published case series.

**Results:**

Cohort comprised 62 children with acquired BMF and 22 children with HAAA. Median age of HAAA patients was 13.5 years. Potentially triggering viral infections were detected in 45%. The median interval from hepatitis onset to cytopenia was 3 weeks. All cases presented with severe hepatitis (median alanine transaminase 2127 U/L) and all but one with hyperbilirubinemia (median bilirubin 15.3 mg/dL). Coagulopathy was variable (median international normalized ratio 1.5). Four patients (18%) developed acute liver failure, two (9%) required liver transplantation. Hepatic parameters normalized within a median of 8.5 weeks. There was no statistically significant difference in the course of hepatitis between patients with SAA and RCC. Early lymphopenia was a key finding in patients with HAAA, progressing from a median of 905/µL at hepatitis onset to 530/µL within 4 weeks.

**Conclusions:**

HAAA occurs in both SAA and RCC. Most cases present with severe acute cholestatic hepatitis and variable coagulopathy. Hepatic recovery is common. Lymphopenia at disease onset is frequent and may serve as a diagnostic marker.

## Abstract (highlights)

**What is Known:**

- Severe aplastic anemia (SAA) may present with acute severe hepatitis, known as hepatitis‐associated aplastic anemia (HAAA).
- Hepatitis may precede SAA by weeks to months, making an early diagnosis of HAAA challenging.

**What is New:**

- Acute severe cholestatic hepatitis with alanine transaminase > 1000 U/L and marked lymphopenia characterize pediatric HAAA.
- HAAA is also seen in myelodysplastic syndrome type refractory cytopenia of childhood (RCC) resembling the course of hepatitis in SAA.
- Early lymphopenia is associated with HAAA but not with isolated acquired BMF and may support early detection of HAAA.

## Graphical abstract

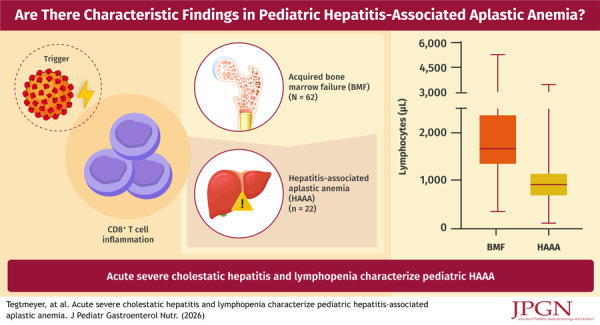

## 1 INTRODUCTION

Acute severe hepatitis of unknown origin, particularly when progressing to pediatric acute liver failure (PALF), is a challenging and potentially life‐threatening condition.^1^, ^2^ Up to 30% have no detectable cause.^1^, ^3^ Of these, 15%–33% develop subsequent bone marrow failure(BMF), commonly reported as hepatitis‐associated aplastic anemia (HAAA).^2^, ^4^, ^5^, ^6^, ^7^ The main acquired BMF syndromes are severe aplastic anaemia (SAA) and myelodysplastic syndrome type refractory cytopenia of childhood (RCC). SAA is considered a CD8‐lymphocellular, autoimmune‐driven BMF, while RCC is of clonal origin.^8^ However, differentiation can be challenging, and an overlapping immune‐mediated pathogenesis has been discussed.^9^ CD8‐driven inflammation has also been observed in liver specimens from HAAA patients indicating a shared immunopathogenesis.^10^, ^11^, ^12^ While hepatitis is well described in SAA, only a few cases in RCC have been reported.^6^, ^13^, ^14^, ^15^, ^16^ Due to its rarity, HAAA has been reported in small pediatric cohorts of 4–15 children.^4^, ^5^, ^6^, ^12^, ^15^, ^16^, ^17^, ^18^, ^19^, ^20^, ^21^, ^22^ Some reported cohorts were drawn from PALF series, although HAAA can occur without coagulopathy.^15^, ^20^ Larger cohorts primarily focused on BMF and provided limited information on the course of hepatitis.^23^, ^24^ Despite the clinical relevance of HAAA, data on the clinical characteristics of the hepatitis are sparse, and no predictor for the occurrence of BMF has been defined. We aimed to characterize the clinical features of pediatric HAAA and analyze available case series to improve understanding of its presentation and course.

## 2 METHODS

### 2.1 Ethics statement

The ethics committees of Hamburg (2022‐100770‐BO‐ff) and Essen (21‐10136‐BO), Germany, approved this scientific project.

### 2.2 Study design and inclusion criteria

Data from all pediatric patients presenting with SAA and RCC at two major German pediatric hematology centers between January 2009 and June 2021 were collected retrospectively. A subgroup of patients with preceding or concomitant hepatitis and thus fulfilling diagnostic criteria for HAAA was pooled and analyzed. The diagnosis of HAAA excluded all patients with evidence of chronic liver disease. PALF is defined according to recent consensus guidelines as acute onset liver disease with no signs of chronic liver disease and international normalized ratio (INR) > 2 not corrected by vitamin K (or INR > 1.5 with encephalopathy).^25^ Lymphocyte counts were also compared with those of SAA and RCC patients without hepatitis. Absolute lymphopenia in children older than 1 year is defined as lymphocyte numbers below 1000/µL.^26^ Patients with preceding hepatitis were first seen by the hepatologist and were later transferred to the hematology division for the initiation of immunosuppression (IST) or hematopoeitic stem cell transplantation (HSCT). SAA and RCC were diagnosed according to published criteria including The European Working Group of MDS and SAA in childhood (EWOG‐SAA) registry and World Health Organization (WHO) classification.^8^, ^27^, ^28^ Onset of cytopenia was defined as significant decline of at least one cell line (hemoglobin [Hb] < 10 g/dL, platelets <100 × 10^3^/µL, absolute neutrophil count [ANC] < 1000/µL). HAAA was defined as alanine transaminase (ALT) > 200 U/L before or at the onset of SAA or RCC. Pre‐existing hepatological diseases were ruled out. In contrast to current WHO criteria of acute severe hepatitis (ALT > 500 U/L), a lower ALT threshold was used to capture potentially milder cases of HAAA.^29^ Hepatic recovery was defined in line with published criteria as ALT and bilirubin <2x the upper limit of normal and regular synthesis.^6^, ^16^ For characterization of HAAA, the medical history, clinical protocols, and blood samples of HAAA patients were analyzed retrospectively.

### 2.3 Statistical analysis

Due to the small cohort size and the nonnormal distribution of most continuous variables, as indicated by the Kolmogorov–Smirnov test, medians were calculated. For statistical analysis, the Mann–Whitney test was used. For categorical data, the chi‐square test (cell count at least 5) and the Fisher's exact test (at least one cell count <5) were used. For correlation analysis, a simple linear regression was performed. A *p*‐value of <0.05 was considered statistically significant (*). Additional significance levels were defined as **p < 0.01, ***p < 0.001, and ****p < 0.0001.

## 3 RESULTS

For this two‐center study, a total of 62 patients with BMF were identified, including 22 with SAA and 40 with RCC Table S1. Of these, 13 (59%) SAA patients and 9 (23%) RCC patients developed acute severe hepatitis before or at the onset of BMF, fulfilling diagnostic criteria for HAAA. The resulting subcohort of 22 HAAA patients was analyzed in detail (Table 1, Table S2).

**Table 1:** Patients' characteristics.

HAAA cohort		Total (*n* = 22)
**Patient characteristics**		
Age (years)	Median (range)	13.5 (3–17)
Male	*n* (%)	13 (59)
Infectious trigger	n (%)	10 (45)
**Laboratory findings**		
ALT (U/L) at maximum	Median (range)	2127 (727–4000)
AST (U/L) at maximum	Median (range)	1823 (237–3530)
GGT (U/L) at maximum	Median (range)	165 (42–810)
Bilirubin (mg/dL) at maximum	Median (range)	15.3 (0.8–28.8)
INR at maximum	Median (range)	1.5 (1.0–4.0)
Lymphocytes (x/µL)^a^ at minimum in Week 1	Median (range)	905 (90–3400)
Lymphocytes (x/µL)^b^ at minimum Weeks 1–4	Median (range)	530 (90–1901)
**Course of disease**		
Hepatitis to BMF (weeks)	Median (range)	3.0 (0–20)
Co‐onset of BMF	*n* (%)	8 (36)
ALF	*n* (%)	4 (18)
LTX	*n* (%)	2 (9)
Hepatic recovery^c^ (weeks)	Median (range)	8.5 (4–25)
Steroid use	*n* (%)	18 (82)
Nonsteroid IST	*n* (%)	16 (73)
HSCT	*n* (%)	16 (73)
Survival^d^	*n* (%)	21 (95)

Abbreviations: ALF, acute liver failure; ALT, alanine transaminase; AST, aspartate aminotransferase; BMF, bone marrow failure; GGT, gamma‐glutamyl transferase; HAAA, hepatitis‐associated aplastic anemia; HSCT, hematopoietic stem cell; INR, international normalized ratio; IST, immunosuppressive treatment; LTX, liver transplantation.

a *n* = 18.

b Three patients with lymphocytes > 1000/µL in first week had no follow‐up data.

^c^ *n* = 20, excluding two patients with LTX.

^d^ Death due to sepsis after HSCT.

Pediatric HAAA occurred at a median age of 13.5 years (range 3–17) with a slight male predominance (59%). All patients were previously healthy. A potential viral trigger was documented in 45%; however, no standardized diagnostic protocol was implemented because of the retrospective study design. Four patients had gastroenteritis (including two cases of rotavirus), five had an upper airway infection (including one case each with respiratory syncytial virus and Epstein‐Barr virus), and one had a herpes simplex virus infection. No genetic abnormalities were found in HAAA patients (Table S1).

The disease course in the 22 HAAA patients was characteristic (Table 1). None of the patients had known prior liver or hematological disease and showed no signs of chronic liver disease or portal hypertension. Median time from hepatitis onset to cytopenia was 3.0 weeks (range 0–20). 36% presented with a simultaneous onset. In all cases, hepatitis presented with an acute and severe onset.

All but one patient presented with jaundice (95%). 27% had abdominal pain, and 45% reported nonspecific symptoms, most frequently fatigue, pallor, or weight loss. Laboratory tests showed markedly elevated ALT with a median of 2127 U/L (range 727–4000) and relatively low gamma‐glutamyl transferase (GGT) with a median of 165 U/L (range 42–810). At presentation, 86% had ALT above 1000 U/L. During the course of the disease, 95% reached ALT > 1000 U/L, and 64% exceeded 2000 U/L. Notably, 59% of patients, including the one with the lowest recorded ALT, reached their peak ALT at disease onset, suggesting that even higher levels may have been missed. Alongside jaundice at presentation, cholestasis was present in all but one patient, with bilirubin reaching a median of 15.3 mg/dL (range 0.8–28.8). The only patient without cholestasis had a mild hepatitis with lower transaminases and rapid hepatic recovery, which was detected 2 weeks before BMF occurred. Hepatic synthetic function was moderately impaired in most cases, reflected by a median INR of 1.5 (range 1.0–4.0) and median albumin of 32 g/L (range 19–41). Criteria for PALF were met in four patients (18%), with a median INR of 2.6 (range 2.3–4.0); two (9%) required urgent liver transplantation (LTX) on Day 5 and Day 15, respectively. The remaining 18 patients (82%) had a median INR of 1.3 (range 1.0–1.8) and no hepatic encephalopathy (HE). Liver biopsies were performed in 16 of the 22 patients. Consistent with the acute pathogenesis of HAAA, all specimens demonstrated lobular and portal inflammation, predominantly CD8‐lymphocellular infiltration, without evidence of advanced fibrosis.

In all but one patient, hepatitis did not relapse. One patient developed a second episode of acute severe hepatitis 7 months later. Laboratory results and the course of hepatitis were similar to the first episode, but this time treated with steroids. Whole exome sequencing for recurrent acute liver failure was performed only in this patient and was negative. After HSCT, no further hepatitis episodes occurred in this patient. All patients exhibited normal levels of immunoglobulin G and no autoantibodies, with the exception of nonspecific low antinuclear antibody (ANA) titers.

**Figure 1:**
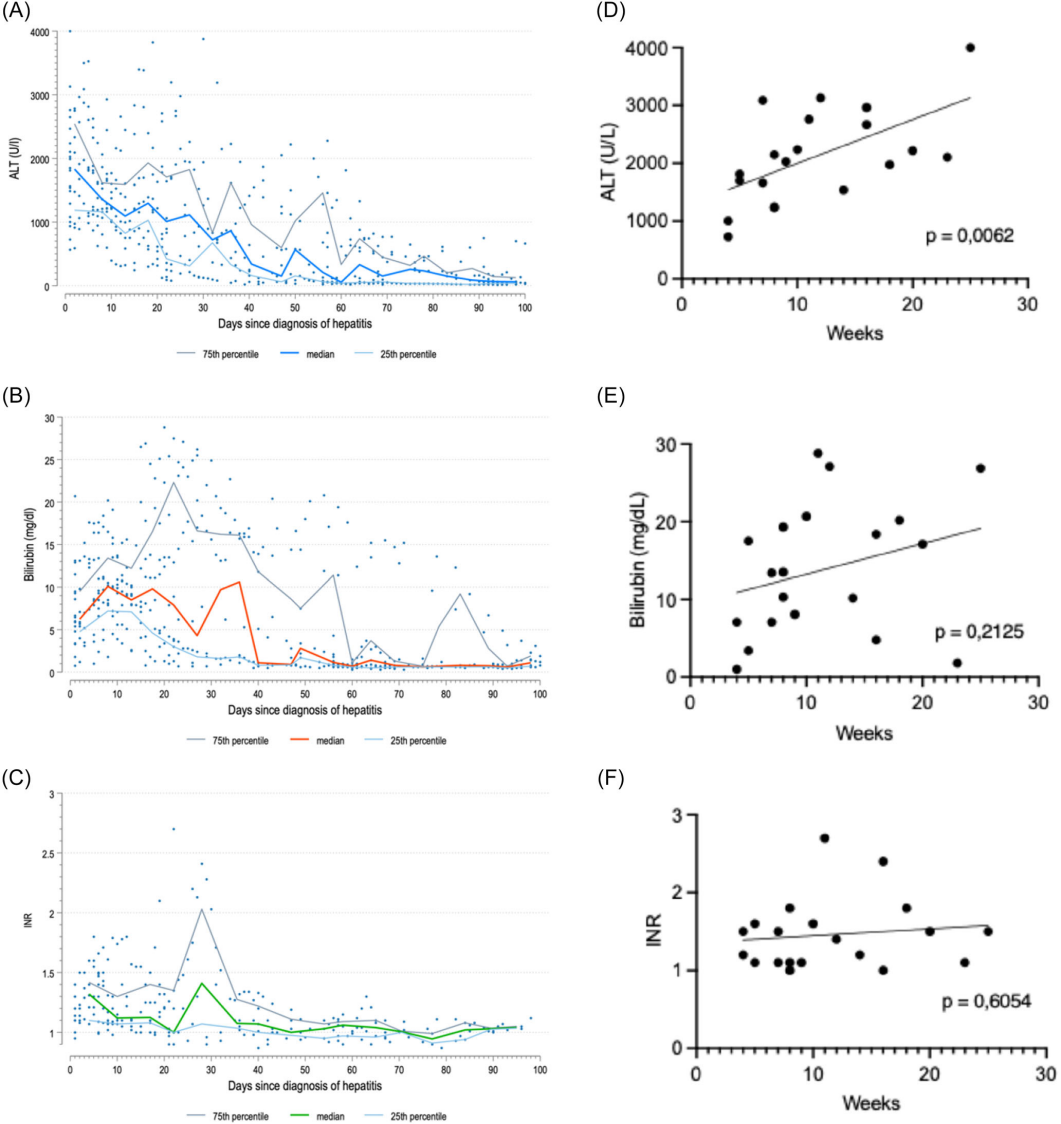
Course of hepatitis. (A–C) Course of ALT, bilirubin, and INR over the first 100 days. Two patients who required urgent liver transplantation were excluded. (D–F) Correlation analysis of peak ALT, bilirubin, and INR and time to hepatic recovery. ALT, alanine transaminase; INR, international normalized ratio.

All cases of hepatitis resolved completely if ALF had not led to early LTX. The course of hepatitis is represented in Figure 1A–C. Liver recovery was observed within a median of 8.5 weeks (4–25) in these 20 cases. Because liver function tests were performed at variable intervals, the time to recovery may be overestimated. The four ALF patients had significantly higher initial median ALT (2706 U/L vs. 1689 U/L, *p* = 0.02). Additionally, peak ALT correlated significantly with the time to full hepatic recovery (*p* = 0.01; Figure 1D). While Figure 1E suggests a possible correlation between bilirubin and time to recovery, statistical significance was not reached due to one patient who, in spite of a prolonged recovery time, had only mild hyperbilirubinemia. INR and thus the occurrence of PALF did not correlate with time to recovery (Figure 1F). A simultaneous onset of hepatitis and BMF did not affect disease severity or course. Co‐onset appeared to be more common in RCC than in SAA (56% vs. 23% respectively), but this difference was not statistically significant (*p* = 0.19). There was also no statistically significant difference in the course of hepatitis between patients with SAA and RCC (Table S3).

Eighteen (82%) children received steroids and other ISTs, including cyclosporine, azathioprine, or anti‐thymocyte‐globulin. Due to small subgroup sizes, treatment effects could not be reliably assessed. Despite the generally favorable hepatic recovery, most patients required HSCT for subsequent BMF (73% overall, 77% in SAA, 67% in RCC). Overall survival was 95%; one patient died from sepsis after HSCT following hepatic recovery.

**Figure 2:**
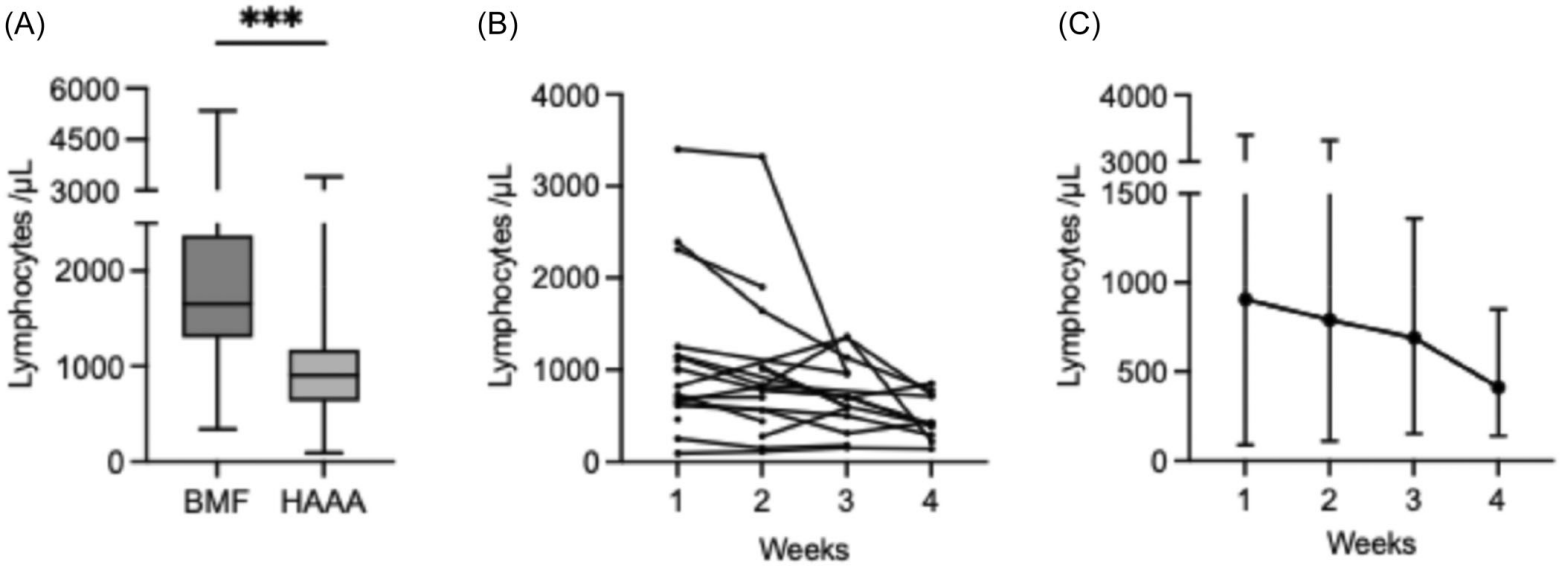
Lymphocyte analysis. (A) Minimum lymphocyte count at disease onset (Week 1), available for BMF (*n* = 29) and HAAA (*n* = 18). Box plot shows the median, interquartiles, and range. ***p = 0.0002. (B) Minimum lymphocyte count during Weeks 1–4. The number of patients with available lymphocyte counts decreased from 18 in Week 1–15 in Weeks 2 and 3, and to 11 in Week 4. (C) Median and range of the individual values shown in (B). BMF, bone marrow failure; HAAA, hepatitis‐associated aplastic anemia.

During the first week of hepatitis, lymphopenia (<1000/µL) was observed in 50% of patients (median 905/µL, range 90–3400), dropping to a median of 530/µL (range 90–1901) within 4 weeks (Figure 2). All patients were acutely ill and showed no signs of chronic liver disease, liver fibrosis, or portal hypertension. Lymphopenia did not correlate with ALT, INR, or potential viral triggers, and was not observed in patients with BMF without hepatitis (median 1650/µL).

## 4 DISCUSSION

This cohort of 22 children with HAAA confirms previously published characteristics of the disease (Table 2). Although an ALT > 200 U/L was defined as the inclusion threshold, all but one patient exceeded 1000 U/L (median 2127 U/L). At onset, most patients, including the one with a maximum ALT of 727 U/L, already had declining transaminases, hinting that even higher levels had been present earlier. Cholestasis with relatively low GGT appears to be a key feature of HAAA. Thus, jaundice was the predominant symptom in all but one patient. Liver biopsies were performed in 16 of 22 patients and showed the previously reported CD8‐dominant inflammation, which is also considered causative for the BMF.^10^, ^11^, ^12^ The acute hepatitis onset is underlined by the fact that no advanced fibrosis or portal hypertension was observed. Highlighting the shared immune‐mediated pathogenesis of SAA and RCC, our cohort demonstrates not only the occurrence of CD8‐dominant hepatitis in both conditions but also a comparable hepatic disease course. Consistent with this mechanism, screened genetic abnormalities were absent in the HAAA subgroup and were detected only in patients with RCC without hepatic injury (Table S1). Their absence in HAAA suggests that genetic lesions primarily affect hematopoietic cells and do not contribute to the immune‐mediated hepatitic injury.

**Table 2:** Cross‐cohort analysis.

**HAAA cohorts**	**Age (Years)**	**Male sex (%)**	**Co‐onset of BMF (%)**	**Lag (w)**	**LTX (%)**	**HSCT (%)**	**ALT (U/L)**	**AST (U/L)**	**Bilirubin (mg/dL)**	**INR**	**Overall survival (%)**
Tegtmeyer et al. (Hamburg and Essen, GER; *n* = 22)	13.5	59	36	3.0	9	77	2127	1823	15.3	1.5	95
Cross‐cohort analysis (16 published cohorts; *n* = 250)	8.3 (*n* = 250)	67 (*n* = 244)	21 (*n* = 191)	4.6 (*n* = 241)	19 (*n* = 250)	26 (*n* = 250)	2980 (*n* = 148)	1609 (*n* = 136)	17.4 (*n* = 143)	2.2 (*n* = 76)	85 (*n* = 250)
Cross‐cohort analysis excluding ALF/LTX‐only cohorts (10 published cohorts; *n* = 196)	**5.8** (*n* = 196)	**65** (*n* = 190)	**27** (*n* = 137)	**4.6** (*n* = 186)	**4** (*n* = 196)	**25** (*n* = 196)	**1767** (*n* = 110)	**1563** (*n* = 100)	**13.3** (*n* = 105)	**1.4** (*n* = 46)	**90** (*n* = 196)

*Note*: 16 case series addressing HAAA were found. Of these, 6 were ALF/LTX‐only cohorts, missing milder forms of HAAA. Cross‐cohort analysis was calculated for continuous data with given medians. If only a mean was reported, a normal distribution of data and a median similar to the mean can be assumed to allow a cross‐cohort analysis.

Abbreviations: ALF, acute liver failure; ALT, alanine transaminase; AST, aspartate aminotransferase; BMF, bone marrow failure; HAAA, hepatitis‐associated aplastic anemia; HSCT, hematopoietic stem cell transplantation; INR, international normalized ratio; LTX, liver transplantation; *n*, number.

Time to hepatic recovery varied, but all patients not requiring urgent LTX fully recovered. The two patients who underwent LTX were diagnosed early in the study period (2010 and 2011). It is possible that growing experience with the generally benign hepatic course in other patients led to more restrictive indications for LTX. Because no treatment protocols are established, each patient was treated individually. Most patients received steroids, but in different doses, at different time points, and with no evidence of a positive effect on the course of hepatitis. Existing data underline this observation.^10^ Case reports suggest the use of anti‐thymocyte globulin (ATG), as it effectively targets the CD8‐driven inflammation in the bone marrow and may also have a beneficial impact on the liver.^6^, ^10^, ^23^, ^30^ However, the effect of IST, and especially ATG, on hepatitis needs to be evaluated in larger, multicenter cohorts.

Early severe lymphopenia seems to be a frequent finding in HAAA. In our cohort lymphopenia did not correlate with onset of BMF, viral infections, or severity of liver disease, most notably PALF. Interestingly, the patients with BMF without hepatitis did not show early lymphopenia, thus pointing to a hepatitis‐specific finding. It remains unclear whether lymphopenia is a specific feature of HAAA or is also associated with other forms of acute severe hepatitis. However, we found no evidence of a similar pattern in other forms of acute hepatitis. Of note, we examined a cohort of autoimmune hepatitis with equally severe onset (ALT > 1000 U/L), who did not present with lymphopenia. Reinforcing these findings, both Maggiore and colleagues and Molina et al. observed lymphopenia in a pediatric hepatitis cohort almost exclusively in the subgroup of associated BMF.^19^, ^21^ The lack of clear diagnostic markers for HAAA, especially in cases with late onset of BMF, underlines the significance of this finding.

Limitations of the study include its retrospective design, non‐standardized diagnostic approach, and small cohort size, which also limited analysis of treatment effects. The described findings were not evaluated for other acute hepatitis entities. Lymphocyte counts were assessed irregularly, and detailed subset analysis was not performed. A potentially adverse prognostic impact of hepatitis on the severity of BMF has been reported, but this was not the focus of our study.^31^, ^32^ By defining BMF as an inclusion criterion, most HAAA patients were likely captured, though asymptomatic cases with complete hepatic recovery before BMF may have been missed. Ten patients had been included in a multicenter study on severe acute hepatitis of unknown origin, but the HAAA subgroup was not separately described.^2^ One patient was previously reported as a case study.^33^

To expand our findings, we reviewed 16 published HAAA cohorts totaling 250 children (Table 2, Table S4). Most cohorts were small and monocentric, had heterogeneous inclusion criteria, and often lacked detailed laboratory data.^4^, ^5^, ^6^, ^7^, ^12^, ^14^, ^15^, ^16^, ^17^, ^18^, ^19^, ^20^, ^21^, ^22^, ^23^, ^24^ Six were drawn from PALF or LTX‐only series, likely missing milder HAAA cases.^4^, ^5^, ^6^, ^17^, ^18^, ^19^ RCC‐associated hepatitis was reported only sporadically.^6^, ^13^, ^14^, ^15^, ^16^ To address these gaps, our two‐center cohort retrospectively included patients with SAA and RCC and defined a relatively low ALT (200 U/L) for inclusion, making it the largest detailed analysis of hepatitis in pediatric HAAA and the first to describe and compare hepatitis in RCC. Osugi et al. and Fu et al. reported two larger cohorts from Japan and China comprising 44 and 81 children, respectively.^23^, ^24^ Both focused on the impact of IST on BMF and lack detailed information on the hepatic course of disease, potentially triggering infections, or early lymphopenia.

Supporting our findings, published cohorts emphasize the characteristic severe onset of HAAA, with a median peak ALT > 2000 U/L and median bilirubin of 17.4 mg/dL. They also show that PALF is not a consistent symptom of HAAA (median peak INR 1.4) and that hepatic recovery is likely, with only 4% requiring LTX.^7^, ^12^, ^14^, ^15^, ^16^, ^20^, ^21^, ^22^, ^23^, ^24^ A hepatic relapse is rare and not described after treatment of BMF. The large number of HSCT in our cohort could be explained by the inclusion of RCC and the highly resourced centers' ability to offer HSCT.

We recommend regular blood monitoring for potential subsequent BMF during the first 6 months after recovery from acute severe hepatitis of unknown origin, as early diagnosis of later BMF may prevent complications such as infection or bleeding and improve treatment response.

The term “HAAA” is closely linked to the subsequent development of BMF, while the hepatitis itself is not independently defined and is often classified in cohorts of “severe acute hepatitis of unknown origin,” “seronegative autoimmune hepatitis,” or “non‐A‐E‐hepatitis.”^2^, ^20^, ^21^ The notable hepatitis‐specific lymphopenia, absent in other forms of acute severe hepatitis or isolated acquired BMF, may reflect a distinct hepatic phenotype.^19^, ^21^ The PALF study group reported frequent “activated CD8 T‐cell hepatitis” in indeterminate PALF, but no data on peripheral lymphocytes were provided.^34^ Evaluating such data could clarify whether CD8‐driven hepatitis in HAAA and indeterminate PALF represents the same entity, potentially supporting a concept of “activated CD8 T‐cell hepatitis” in HAAA as an independent condition rather than solely a precursor to BMF. Also, the shared CD8‐driven inflammation in both SAA and RCC may suggest a shared but independent immunodysregulatory pathogenesis.

Future studies on HAAA and isolated acute severe hepatitis of unknown origin are encouraged to include the lymphocytic phenotype, and if possible, lymphocyte subpopulations in blood and liver samples. This may not only better define HAAA but also help to understand the pathogenesis of HAAA. Future studies should clarify the role of IST, particularly ATG, in improving hepatic recovery and preventing progression to BMF.

## 5 CONCLUSIONS

In conclusion, early diagnosis of HAAA is difficult but may be facilitated by the characteristic pattern of HAAA including extraordinarily high and early peaking transaminases (>1000 U/L), clinically significant cholestasis despite relatively low GGT and often moderate coagulopathy. In particular, lymphopenia at the onset of hepatitis should raise suspicion for the development of associated BMF and should prompt regular monitoring of blood counts during the first months after discharge.

## CONFLICT OF INTEREST STATEMENT

The authors declare no conflicts of interest.

## Supporting information

Supporting information. Supplementary Table 1: Characteristics of all patients with BMF. *Screening for genetic abnormalities included paroxysmal nocturnal hemoglobinuria (PNH), Fanconi anemia, chromosomal abnormalities, and GATA2 mutations. Identified abnormalities were: monosomy 7 (n = 4), trisomy 7 (n = 1), partial deletion of chromosome 7 (n = 1), trisomy 8 (n = 2), PNH clone (n = 6), and GATA2 mutation (n = 3). # With hepatitis n = 21; + With hepatitis n = 18, Without hepatitis n = 29. *Abbreviations*: BMF, bone marrow failure; HSCT, hematopoietic stem cell transplantation.

Supporting information. Supplementary Table 2: Individual patient's characteristics. O, onset; lymphocyte at onset (minimum in week 1) and at minimum (minimum within weeks 1–4). *Patient 2 developed a second episode of acute severe hepatitis 7 months later. Laboratory results and the course of the second hepatitis were similar to the first episode, though treated with steroids. Whole exome sequencing to address genetic recurrend acute liver failure was negative. After hematopoetic stem cell transplantation (HSCT) no further hepatitis episodes occurred. *Abbreviations*: ALF, acute liver failure; ALT, alanine transaminase; AST, aspartate aminotransferase; ATG, anti‐thymocyte globulin; BMF, bone marrow failure; EBV, Epstein‐Barr virus; GE, gastroenteritis; GGT, gamma‐glutamyl transferase; HSV, herpes simplex virus; ICH, intracranial hemorrhage; ICU, intensive care unit; INR, international normalized ratio; LTX, liver transplantation; RCC, refractory cytopenia of childhood; RI, respiratory infection; RSV, respiratory syncytial virus; RV, rotavirus; saA, severe aplastic anemia.

Supporting information. Supplementary Table 3: Comparison of SAA and RCC. *n = 18 (SAA n = 12, RCC n = 6); **Three patients with lymphocytes > 1000/µL in first week had no follow up data. ^+^n = 20, excluding two patients with LTX. #Death due to sepsis after HSCT. *Abbreviations*: ALF, acute liver failure; ALT, alanine transaminase; AST, aspartate aminotransferase; BMF, bone marrow failure; GGT, gamma‐glutamyl transferase; HSTC, hematopoietic stem cell transplantation; INR, international normalized ratio; IST, immunosuppressive treatment; LTX, liver transplantation; RCC, refractory cytopenia of childhood; saA, severe aplastic anemia.

Supporting information. Supplementary Table 4: Analysis of published cohorts. “2 patients with ‘transient bone marrow suppression’ not fulfilling aplastic anemia (AA) criteria were excluded. Of the remaining 6 patients, 2 tested positive for parvo‐B19 virus. ^M^Mean. *Number is approximated by given information. **5 patients with “mild bone marrow hypoplasia” not fulfilling AA criteria and 2 patients with confirmed GALD were excluded. ***The cohort overlaps with Tung et. al 5 years, recalling 3 patients that were excluded. Also, 4 with “transient bone marrow suppression” not fulfilling AA criteria were excluded. ****Conjugated bilirubin. ****Analysis was only performed for 5 patients with RCC. ## Of initially 18 patients, 15 were reported with liver enzyme tests. *Abbreviations*: ALT, alanine transaminase; AST, aspartate aminotransferase; ATG, anti‐thymocyte globulin; BMF, bone marrow failure; HAAA, hepatitis‐associated aplastic anemia; HSTC, hematopoietic stem cell transplantation; ICH, intracranial hemorrhage; LTX, liver transplantation; mo, months; M, mean; Mdn., median; RCC, refractory cytopenia of childhood; NA, not available; saA, severe aplastic anemia; VOD, veno‐occlusive disease; w, weeks.
