# Supplementary material for: Acute severe cholestatic hepatitis and lymphopenia characterize pediatric hepatitis‐associated aplastic anemia
Source: J Pediatr Gastroenterol Nutr. 2025 Dec 9;82(2):374–82. doi: 10.1002/jpn3.70308 (PMC12864175; doi:10.1002/jpn3.70308)
Supplement: Supplementary file 1 — Supporting information. Supplementary Table 1: Characteristics of all patients with BMF. *Screening for genetic abnormalities included paroxysmal nocturnal hemoglobinuria (PNH), Fanconi anemia, chromosomal abnormalities, and GATA2 mutations. Identified abnormalities were: monosomy 7 (n = 4), trisomy 7 (n = 1), partial deletion of chromosome 7 (n = 1), trisomy 8 (n = 2), PNH clone (n = 6), and GATA2 mutation (n = 3). # With hepatitis n = 21; + With hepatitis n = 18, Without hepatitis n = 29. Abbreviations: BMF, bone marrow failure; HSCT, hematopoietic stem cell transplantation. [file JPN3-82-374-s004.docx]

**Supplementary Table 1: Characteristics of all patients with BMF**

| **BMF Cohort** |  | **Total**  **(n = 62)** | **With hepatitis**  **(n = 22)** | **No hepatitis**  **(n = 40)** | **p-value** |
| --- | --- | --- | --- | --- | --- |
| **Patient characteristics** | | | | | |
| **Age (years)** | median  (range) | **10.5**  (2–17) | 13.5  (3–17) | 9.0  (2–17) | 0.24 |
| **Male** | n (%) | **39 (63)** | 13 (59) | 26 (65) | 0.64 |
| **Infectious trigger** | n (%) | **20 (32)** | 10 (45) | 10 (25) | 0.60 |
| **Genetic abnormalities*** | n (%) | **15 (24)** | 0 (0) | 15 (38) | **0.002** |
| **Laboratory findings** | | | | | |
| **Hemoglobin (g/dL)**^#^  at minimum | median  (range) | **6.7**  (3.7–13.1) | 6.7  (4.7–12.3) | 6.7  (3.7–13.1) | 0.74 |
| **Absolute neutrophil**^#^ **count (x/µL)**  at minimum | median  (range) | **150**  (0–1650) | 90  (0–1650) | 200  (0–1630) | 0.41 |
| **Platelets (x/µL)**^#^  at minimum | median  (range) | **8**  (0–170) | 5  (0–54) | 9  (0–170) | 0.10 |
| **Lymphocytes (x/µL)**^+^  at minimum in week 1 | median  (range) | **1310**  (90–5340) | **905**  (90–3400) | 1650  (740–5340) | **0.0002** |
| **Course of disease** | | | | | |
| **HSCT** | n (%) | **51 (82)** | 16 (73) | 34 (85) | 0.32 |
