# Supplementary material for: Acute severe cholestatic hepatitis and lymphopenia characterize pediatric hepatitis‐associated aplastic anemia
Source: J Pediatr Gastroenterol Nutr. 2025 Dec 9;82(2):374–82. doi: 10.1002/jpn3.70308 (PMC12864175; doi:10.1002/jpn3.70308)
Supplement: Supplementary file 2 — Supporting information. Supplementary Table 2: Individual patient's characteristics. O, onset; lymphocyte at onset (minimum in week 1) and at minimum (minimum within weeks 1–4). *Patient 2 developed a second episode of acute severe hepatitis 7 months later. Laboratory results and the course of the second hepatitis were similar to the first episode, though treated with steroids. Whole exome sequencing to address genetic recurrend acute liver failure was negative. After hematopoetic stem cell transplantation (HSCT) no further hepatitis episodes occurred. Abbreviations: ALF, acute liver failure; ALT, alanine transaminase; AST, aspartate aminotransferase; ATG, anti‐thymocyte globulin; BMF, bone marrow failure; EBV, Epstein‐Barr virus; GE, gastroenteritis; GGT, gamma‐glutamyl transferase; HSV, herpes simplex virus; ICH, intracranial hemorrhage; ICU, intensive care unit; INR, international normalized ratio; LTX, liver transplantation; RCC, refractory cytopenia of childhood; RI, respiratory infection; RSV, respiratory syncytial virus; RV, rotavirus; saA, severe aplastic anemia. [file JPN3-82-374-s001.docx]

**Supplementary Table 2: Individual patient’s characteristics**

| **No.** | **BMF type** | **Age**  **(y)** | **Sex** | **Trigger** | **Symptoms** | **Lag**  **(w)** | **AST**  **(U/L)** | | **ALT**  **(U/L)** | | **GGT**  **(U/L)** | | **Bili**  **(mg/dL)** | | **INR** | | **Lymphocytes (x/µL)** | | **LTX** | **Recovery**  **(w)** | **Steroid use for hepatitis** | **Treatment of BMF** |
| --- | --- | --- | --- | --- | --- | --- | --- | --- | --- | --- | --- | --- | --- | --- | --- | --- | --- | --- | --- | --- | --- | --- |
|  |  |  |  |  |  |  | **O** | **Max** | **O** | **Max** | **O** | **Max** | **O** | **Max** | **O** | **Max** | **O** | **Min** |  |  |  |  |
| **1** | RCC | 14 | m | no | jaundice, abdominal pain | 2 | 1680 | 3530 | 3130 | 3130 | 145 | 145 | 5.9 | 27.1 | 1.2 | 1.4 | na | 380 | no | 12 | yes | azathioprine, watch and wait |
| **2** | RCC | 6 | m | no | jaundice, abdominal pain, fatigue | 0 | 823 | 2802 | 2237 | 2237 | 76 | 76 | 20.7 | 20.7 | 1.6 | 1.6 | 700 | 700 | no | 10 | no | HSCT |
| **3** | RCC | 16 | m | RI with RSV | jaundice | 4 | 1614 | 2537 | 4000 | 4000 | 86 | 810 | 9.3 | 26.9 | 1.3 | 1.5 | 660 | 220 | no | 25 | yes | cyclosporine, sirolimus, HSCT |
| **4** | RCC | 15 | f | GE with RV | jaundice, nausea, vomiting | 6 | 565 | 1323 | 1081 | 1663 | 67 | 81 | 3.3 | 7.1 | 1.0 | 1.1 | na | 600 | no | 7 | yes | HSCT |
| **5** | RCC | 16 | m | no | jaundice | 0 | 1060 | 1703 | 937 | 1240 | 208 | 301 | 5.9 | 19.3 | 1.0 | 1.0 | 610 | 310 | no | 8 | yes | cyclosporine, HSCT |
| **6** | SAA | 5 | m | RI with EBV | jaundice, abdominal pain | 0 | 1889 | 1889 | 2105 | 2105 | 261 | 393 | 1.8 | 1.8 | 1.1 | 1.1 | 990 | 990 | no | 23 | no | HSCT |
| **7** | SAA | 16 | m | no | jaundice, fatigue | 18 | 1250 | 1250 | 2958 | 2958 | 42 | 42 | 26.7 | 26.7 | 1.9 | 2.3 | 1100 | 1100 | yes | LTX | no | ATG, cyclosporine, steroids, HSCT |
| **8** | SAA | 17 | f | no | jaundice, fatigue | 20 | 1435 | 1510 | 1488 | 1975 | 35 | 150 | 13.1 | 20.2 | 1.4 | 1.8 | 1010 | 410 | no | 18 | yes | azathioprine, HSCT |
| **9** | SAA | 7 | m | RI | jaundice, abdominal pain | 0 | 1823 | 1823 | 2027 | 2027 | 85 | 253 | 8.1 | 8.1 | 1.1 | 1.1 | na | 277 | no | 9 | yes | cyclosporine, steroids, HSCT |
| **10** | SAA | 14 | m | no | jaundice, vomiting, weight loss, fatigue | 4 | 1205 | 1205 | 1003 | 1003 | 164 | 208 | 6.0. | 7.1 | 1.5 | 1.5 | 2309 | 1901 | no | 4 | no | HSCT |
| **11** | SAA | 15 | f | GE | jaundice, fatigue, nausea | 2 | 1870 | 2187 | 2761 | 2761 | 45 | 217 | 4.0 | 28.8 | 1.2 | 2.7 | 2390 | 780 | no | 11 | yes | HSCT |
| **12** | SAA | 13 | f | ICH / ICU care | none | 2 | 237 | 237 | 727 | 727 | 88 | 88 | 0.8 | 1.0 | na | 1.2 | 640 | 290 | no | 4 | yes | ATG, cyclosporine, steroids, HSCT |
| **13** | SAA | 5 | f | HSV | jaundice, abdominal pain | 4 | 1515 | 1839 | 1992 | 2149 | 105 | 143 | 6.7 | 13.5 | 1.3 | 1.8 | 1251 | 960 | no | 8 | yes | HSCT |
| **14** | RCC | 15 | f | no | jaundice, abdominal pain, fatigue | 5 | 866 | 1621 | 1291 | 1699 | 149 | 169 | 6.5 | 17.5 | 1.4 | 1.6 | 3400 | 960 | no | 5 | yes | ATG, cyclosporine, steroids |
| **15** | SAA | 3 | f | RI | jaundice, vomiting | 4 | 2114 | 2984 | 2326 | 2326 | 252 | 252 | 10.8 | 17.1 | 1.3 | 4.0 | 820 | 730 | yes | LTX | yes | ATG, cyclosporine (tacrolimus post-LTX), steroids |
| **16** | SAA | 12 | f | no | jaundice | 8 | 461 | 994 | 591 | 1231 | 81 | 82 | 9.5 | 10.3 | 1.1 | 1.1 | 730 | 440 | no | 8 | yes | ATG, cyclosporine, steroids, HSCT |
| **17** | SAA | 16 | m | no | jaundice, weight loss | 7 | 1402 | 1402 | 1813 | 1813 | 143 | 160 | 3.4 | 3.4 | 1.1 | 1.1 | 460 | 460 | no | 5 | yes | ATG, cyclosporine, steroids |
| **18** | SAA | 15 | m | no | jaundice | 4 | 1033 | na | 1860 | 2668 | 65 | 65 | 4.8 | 4.8 | 1.0 | 1.0 | 1140 | 710 | no | 7 | yes | ATG, cyclosporine, steroids, HSCT |
| **19** | SAA | 5 | m | GE with RV | jaundice, nausea, fatigue | 0 | 1799 | 3080 | 1565 | 2219 | 90 | 97 | 12.9 | 17.1 | 1.2 | 1.5 | 1150 | 690 | no | 20 | yes | ATG, cyclosporine, steroids |
| **20** | RCC | 11 | m | GE | jaundice, fatigue | 0 | 2626 | 3261 | 2468 | 3089 | 157 | 299 | 11.5 | 13.4 | 1.3 | 1.5 | 250 | 150 | no | 7 | yes | ATG, cyclosporine, steroids, HSCT |
| **21** | RCC | 8 | f | no | jaundice addominal pain | 0 | 705 | 842 | 1539 | 1539 | 152 | 361 | 8.6 | 10.2 | 1.1 | 1.2 | 90 | 90 | no | 14 | yes | ATG, cyclosporine, steroids, HSCT |
| **22** | RCC | 6 | m | RI | jaundice, fatigue | 0 | 2025 | 2501 | 2651 | 2966 | 114 | 289 | 5.4 | 18.4 | 1.1 | 2.4 | na | 412 | no | 16 | yes | ATG, cyclosporine, steroids |
