# Supplementary material for: Acute severe cholestatic hepatitis and lymphopenia characterize pediatric hepatitis‐associated aplastic anemia
Source: J Pediatr Gastroenterol Nutr. 2025 Dec 9;82(2):374–82. doi: 10.1002/jpn3.70308 (PMC12864175; doi:10.1002/jpn3.70308)
Supplement: Supplementary file 3 — Supporting information. Supplementary Table 3: Comparison of SAA and RCC. *n = 18 (SAA n = 12, RCC n = 6); **Three patients with lymphocytes > 1000/µL in first week had no follow up data. +n = 20, excluding two patients with LTX. #Death due to sepsis after HSCT. Abbreviations: ALF, acute liver failure; ALT, alanine transaminase; AST, aspartate aminotransferase; BMF, bone marrow failure; GGT, gamma‐glutamyl transferase; HSTC, hematopoietic stem cell transplantation; INR, international normalized ratio; IST, immunosuppressive treatment; LTX, liver transplantation; RCC, refractory cytopenia of childhood; saA, severe aplastic anemia. [file JPN3-82-374-s002.docx]

**Supplementary Table 3: Comparison of SAA and RCC**

| **HAAA Cohort** |  | **Total**  **(n = 22)** | **SAA**  **(n = 13)** | **RCC**  **(n = 9)** | **p-value** |
| --- | --- | --- | --- | --- | --- |
| **Patient characteristics** | | | | | |
| **Age (years)** | median  (range) | **13.5**  (3–17) | 13.0  (3–17) | 14.0  (6–16) | 0.66 |
| **Male** | n (%) | **13 (59)** | 7 (54) | 6 (67) | 0.67 |
| **Infectious trigger** | n (%) | **10 (45)** | 6 (46) | 4 (44) | > 0.99 |
| **Laboratory findings** | | | | | |
| **ALT (U/L)** at maximum | median  (range) | **2127**  (727–4000) | 2105  (727–2958) | 2237  (1240–4000) | 0.26 |
| **AST (U/L)**  at maximum | median  (range) | **1823**  (237–3530) | 1667  (237–3080) | 2501  (842–3530) | 0.25 |
| **GGT (U/L)**  at maximum | median  (range) | **165**  (42–810) | 150  (42–393) | 289  (81–810) | 0.19 |
| **Bilirubin (mg/dL)**  at maximum | median  (range) | **15.3**  (0.8–28.8) | 10.3  (0.8–28.8) | 18.4  (7.1–27.1) | 0.11 |
| **INR**  at maximum | median  (range) | **1.5**  (1.0–4.0) | 1.5  (1.0–4.0) | 1.5  (1.0–2.4) | 0.88 |
| **Lymphocytes (x/µL)***  at minimum in week 1 | median  (range) | **905**  (90–3400) | 1055  (460–2390) | 635  (90–3400) | 0.08 |
| **Lymphocytes (x/µL)****  at minimum week 1-4 week** | median  (range) | **530**  (90–1901) | 710  (277–1901) | 380  (90–960) | **0.04** |
| **Course of disease** | | | | | |
| **Hepatitis to BMF (weeks)** | median  (range) | **3.0**  (0–20) | 4.0  (0–20) | 0  (0–6) | 0.14 |
| **Co-onset of BMF** | n (%) | **8 (36)** | 3 (23) | 5 (56) | 0.19 |
| **ALF** | n (%) | **4 (18)** | 3 (23) | 1 (11) | 0.62 |
| **LTX** | n (%) | **2 (9)** | 2 (15) | 0 (0) | 0.49 |
| **Hepatic recovery**^+^ **(weeks)** | median  (range) | **8.5**  (4–25) | 8.0  (4–23) | 10.0  (5–25) | 0.61 |
| **Steroids use** | n (%) | **18 (82)** | 10 (77) | 8 (89) | 0.14 |
| **Non-steroid IST** | n (%) | **16 (73)** | 9 (69) | 7 (78) | > 0.99 |
| **HSCT** | n (%) | **16 (73)** | 10 (77) | 6 (67) | 0.66 |
| **Survival**^#^ | n (%) | **21** **(95)** | 12 (92) | 9 (100) | > 0.99 |
