# Supplementary material for: Acute severe cholestatic hepatitis and lymphopenia characterize pediatric hepatitis‐associated aplastic anemia
Source: J Pediatr Gastroenterol Nutr. 2025 Dec 9;82(2):374–82. doi: 10.1002/jpn3.70308 (PMC12864175; doi:10.1002/jpn3.70308)
Supplement: Supplementary file 4 — Supporting information. Supplementary Table 4: Analysis of published cohorts. “2 patients with ‘transient bone marrow suppression’ not fulfilling aplastic anemia (AA) criteria were excluded. Of the remaining 6 patients, 2 tested positive for parvo‐B19 virus. MMean. *Number is approximated by given information. **5 patients with “mild bone marrow hypoplasia” not fulfilling AA criteria and 2 patients with confirmed GALD were excluded. ***The cohort overlaps with Tung et. al 5 years, recalling 3 patients that were excluded. Also, 4 with “transient bone marrow suppression” not fulfilling AA criteria were excluded. ****Conjugated bilirubin. ****Analysis was only performed for 5 patients with RCC. ## Of initially 18 patients, 15 were reported with liver enzyme tests. Abbreviations: ALT, alanine transaminase; AST, aspartate aminotransferase; ATG, anti‐thymocyte globulin; BMF, bone marrow failure; HAAA, hepatitis‐associated aplastic anemia; HSTC, hematopoietic stem cell transplantation; ICH, intracranial hemorrhage; LTX, liver transplantation; mo, months; M, mean; Mdn., median; RCC, refractory cytopenia of childhood; NA, not available; saA, severe aplastic anemia; VOD, veno‐occlusive disease; w, weeks. [file JPN3-82-374-s003.docx]

**Supplementary Table 4: Analysis of published cohorts**

| **Publication** | **Year** | **Recruiting cohort** | **Pediatric HAAA cohort** | **Age (y)** | **Male sex** | **Co-onset**  **of AA** | **Lag (w)** | **LTX** | **HSCT** | **ALT (U/L)** | **AST (U/L)** | **Bilirubin (mg/dL)** | **INR** | **Hepatic course of disease** | **Overall survival** |
| --- | --- | --- | --- | --- | --- | --- | --- | --- | --- | --- | --- | --- | --- | --- | --- |
|  |  |  | N | Mdn.  (range) | % | N (%) | Mdn.  (range) | N (%) | N (%) | Mdn.  (range) | Mdn.  (range) | Mdn.  (range) | Mdn.  (range) |  |  |
| **Tzakis et al**.  (Pittsburgh, USA) | 1988 | Pediatric LTX in idiosyncratic ALF (n = 32), single center, until 1987 | 8 | 7.0  (3 - 14) | 75 | 0 (0) | 10  (3 - 22) | 8 (100) | 0 (0) | 24480  (5280 - 161100) | NA | 34.0  (22.1 - 38.6) | NA | All 8 LTX | 63 %  (3 deaths: 3 fungal infections) |
| **Cattral et al**.  (Omaha, USA) | 1994 | Pediatric LTX (n = 18), single center,  1985 - 1993 | 6 | 8.0  (1,2-16) | 83 | 0 (0) | 4  (1 - 7) | 6 (100) | 0 (0) | 1196  (174 - 2459) | 903  (115 - 4625) | 27.4  (21.1 - 34.0) | NA | All 6 LTX | 50 %  (3 death post-LTXs: 2 fungal sepsis, 1 ICH) |
| **Brown et al**.  (Bethesda, USA) | 1997 | HAAA (n = 10),  single center,  1990 - 1996 | 5 | 15.0  (8 - 18) | 80 | 1 (20) | 5  (0 - 7) | 0 (0) | 1 (20) | 1727  (527 - 3154) | 2228  (1131 - 4517) | NA | NA | All 5 full hepatic recovery (4 within 1 mo and 1 after relapse) | 60 %  (2 deaths: 1 post-HSCT fungal sepsis, 1 post-HSCT VOD) |
| **Tung et al**.  (London, UK) | 2000 | Pediatric ALF (n = 75),  single center,  1975 - 1998 | 6” | 4.3  (3 - 11) | 50 | 0 (0) | 4.9  (3 - 14) | 4 (67) | 2 (33) | NA | 827  (430 - 11000) | NA  (“jaundice”) | 3.2  (1.3 - 6.8) | 4 LTX,  2 “improved” | 50 %  (3 deaths: 1 sepsis post-HSCT, 1 BMF, 1 leukemia) |
| **Ohara et al**.  (Tokyo, JP) | 2022 | Pediatric acquired BMF (n = 525),  multi center registry, 1988 - 1996 | 5**** | 11.0  (4-13) | 40 | 0 (0) | 8.5  (6 – 9) | 0 (0) | 3 (60) | 1143  (923 – 2050) | 1183  (673 – 1360) | 22.5  (3.5 – 25.6) | NA | No specific information, hepatic recovery assumed | 20 % (4 deaths: MDS-associated) |
| **Molina et al.**  (Los Angeles, USA) | 2004 | Pediatric ALF (n = 110), single center,  1985 - 2003 | 9 | 4.8  (1.3 - 14) | 100 | 0 (0) | 5.1  (2 - 22) | 9 (100) | 2 (22) | 1305^M^  (“+/-160”) | 1598^M^  (“+/-293”) | 20.0^M^  (“+/-2,5”) | 2.3^M^*  (2.0 - 2.7) | All 9 LTX | 71%  (2 deaths post-LTX: 1 multiorgan failure, 1 GvHD) |
| **Osugi et al.**  (Osaka, JP) | 2007 | Pediatric SAA (n = 319),  multi center AA registry, 1992 - 2001 | 44 | 9.0  (1 - 18) | 64 | NA | 4  (0 - 34) | 0 (0) | 2 (5) | 1181  (521 - 2570) | 1012  (200 - 2893) | 8.8  (1.1 - 23.9) | NA | No specific information, hepatic recovery assumable | 91%  (4 deaths of infection) |
| **Honkaniemi et al.**  (Stockholm, SWE) | 2007 | Pediatric HAAA,  single center,  1988 - 2006 | 7 | 10.0  (2.5 - 15) | 57 | 1 (14) | 7.7  (0 - 18) | 1 (14) | 3 (43) | 2760  (1122 - 6660) | 2700  (1260 - 7500) | 18.8  (3.7 - 45.2) | 1.6  (1.2 - 3.2) | 1 LTX, 1 died of ALF, 1 recovered, 4 treated with ATG or HSCT but hepatitis had improved before) | 86%  (1 death of sepsis during ALF) |
| **Hadzic et al.**  (London, UK)*** | 2008 | Idiosyncratic pediatric ALF (n = 96), single center,  1989 - 2005 | 10*** | 6.8  (0.8 - 14) | 90 | 1 (10) | 6.0  (2 - 17) | 3 (30) | 5 (50) | NA | NA | NA | NA | 3 LTX, 6 hepatic recovery (4 within 2 mo, 1 within 22 mo, 2 not specified), 1 death from sepsis in ALF | 80%  (2 deaths: 1 sepsis in ALF, 1 post-LTX multi organ failure) |
| **Tschiedel et al.**  (Essen, GER) | 2010 | Pediatric HAAA, single center,  1999 - 2008 | 6 | 8.5  (0.8 - 11) | NA | 1 (17) | 6.9  (0 - 8) | 0 (0) | 4 (67) | 1770  (1147 - 2821) | 1913  (1259 - 2590) | 13.9  (2.5 - 27.5) | 1.2  (1.0 - 1.7) | All 6 with full hepatic recovery after mean 10.8 (4-24) weeks | 83%  (1 death: acute post-HSCT complications) |
| **Maggiore et al.**  (Pisa, IT and Paris, FR) | 2016 | Pediatric hepatitis (n = 38), two centers,  1988 - 2010 | 10 | 11.0  (9 - 14) | 50 | NA | NA | 1 (10) | 0 (0) | 2250  (1395 - 4500) | NA | 26.5  (4.6 - 43.2) | NA | 1 LTX, 9 with full hepatic recovery median 8 w (ca. 6-9) | 100% |
| **Patel et al.**  (Houston, USA) | 2017 | Pediatric HAAA,  single center,  2009 - 2014 | 7 | 7.5  (4 - 17) | 71 | 3 (43) | 5.0  (0 - 42) | 2 (29) | 3 (43) | ﻿ 2545  (910 - 3395) | ﻿ 1775  (1507 - 3976) | 7.5  (4.0 - 16.6) | 1.2  (1.1 - 4.0) | 2 LTX, 5 “improved”, (incl. 1 relapse after cessation of ATG) | 86%  (1 death: post HSCT sepsis) |
| **Böske et al**.  (Hanover, GER) | 2020 | LTX in idiosyncratic pediatric ALF (n = 42, additional cases  (n = 7), single center,  1984 - 2017 | 15^##^ | 8.4  (1.4 - 16) | 50 | 2 (11) | 1.0  (0 - 15) | 11 (61) | 6 (33) | 2130  (462 - 3179) | 2518  (1728 - 4581) | 32.0  (8.7 - 41.1) | 4.0*  (1.2 - 10.0) | 11 LTX (incl. 2 who died peri-LTX from sepsis and ICH),  6 “remission”, 1 death from ALF | 67%  (6 deaths: 1 ALF, 1 post-LTX sepsis, 1 post-LTX ICH, 1 pre-HSCT sepsis, 2 post-HSCT sepsis |
| **Kemme et al.**  (Denver, USA) | 2021 | Pediatric HAAA,  single center,  2016 - 2019 | 4 | 6.5  (5 - 7) | 75 | 3 (75) | 7^M^*  (0 - 16) | 0 (0) | 2 (50) | 2633  (2445 - 2763) | 2962  (2287 - 3485) | 7.5  (6.7 - 10.7) | 1.1  (1.0 - 1.5) | All 4 with full hepatic recovery after mean 15 w*(12-24) | 100% |
| **Mehta et al.**  (Toronto, Canada) | 2023 | Pediatric HAAA,  single center,  2018 - 2022 | 5 | 8.2  (1.3 – 13.9) | 60 | NA | 8.6  (0 – 22.3) | 1 (20) | 3 (60) | NA | NA | NA | NA | 2 ALF, 1 LTX | 100% |
| **Fu et al.**  (Beijing, China) | 2024 | Pediatric aplastic anemia  (n = 910), single center,  2017 - 2021 | 81 | 6.0  (1.0 - 13.9) | 69 | 20 (27) | 4.2  (0 - 30) | 0 (0) | 11 (14) | NA | NA | NA | NA | At onset 31 (38%) with ALT 200-500 U/L, 50 (62%) with ALT > 500 U/L, 4 ALF, no LTX | 94%  (1 infection, 4 intracranial hemorrhage) |
| **Tegtmeyer et al.**  (Hamburg and Essen, GER) | 2025 | Pediatric BMF (SAA and MDS-RCC) (n = 62), two centers,  2009 - 2021 | 22 | 13.5  (3 - 17) | 59 | 8 (36) | 3.0  (0 - 20) | 2 (9) | 17 (77) | 2127  (727 - 4000) | 1823  (237 - 3530) | 15.3  (0.8 - 28.8) | 1.5  (1.0 – 4.0) | 4 ALF, 2 LTX, 20 with full hepatic recovery after median 8.5 w (4-25) | 95%  (1 death: post-HSCT sepsis) |
